# Abnormal energy regulation in early life: childhood gene expression may predict subsequent chronic mountain sickness

**DOI:** 10.1186/1471-2431-8-47

**Published:** 2008-10-27

**Authors:** Luis Huicho, Guoqiang Xing, Clifford Qualls, María Rivera-Ch, Jorge L Gamboa, Ajay Verma, Otto Appenzeller

**Affiliations:** 1 Departament of Paediatrics, Universidad Nacional Mayor de San Marcos, Universidad Peruana Cayetano Heredia, and Instituto de Salud del Niño, Lima, Peru; 2 Department of Psychiatry, Uniformed Services University of the Health Sciences, Bethesda MD, USA; 3 University of New Mexico, Department of Mathematics and Statistics and Clinical Research Center, University of New Mexico School of Medicine, Albuquerque NM 887131, USA; 4 Departament of Biological and Physiological Sciences, Faculty of Sciences and Philosophy, High Altitude Research Institute, Universidad Peruana Cayetano Heredia, Lima, Peru; 5 Department of Physiology, University of Kentucky, Lexington, Kentucky, USA; 6 Department of Neurology, Uniformed Services University of the Health Sciences, Bethesda MD, USA; 7 Department of Neurology, New Mexico Health Enhancement and Marathon Clinics Research Foundation, Albuquerque NM, USA

## Abstract

**Background:**

Life at altitude depends on adaptation to ambient hypoxia. In the Andes, susceptibility to chronic mountain sickness (CMS), a clinical condition that occurs to native highlanders or to sea level natives with prolonged residence at high altitude, remains poorly understood. We hypothesized that hypoxia-associated gene expression in children of men with CMS might identify markers that predict the development of CMS in adults. We assessed distinct patterns of gene expression of hypoxia-responsive genes in children of highland Andean men, with and without CMS.

**Methods:**

We compared molecular signatures in children of highland (HA) men with CMS (n = 10), without CMS (n = 10) and in sea level (SL) children (n = 20). Haemoglobin, haematocrit, and oxygen saturation were measured. Gene expression in white cells was assessed at HA and then, in the same subjects, within one hour of arrival at sea level.

**Results:**

HA children showed higher expression levels of genes regulated by HIF (hypoxia inducible factor) and lower levels of those involved in glycolysis and in the tricarboxilic acid (TCA) cycle. Pyruvate dehydrogenase kinase 1(PDK1) and HIF prolyl hydroxylase 3 (HPH3) mRNA expressions were lowest in children of CMS fathers at altitude. At sea level the pattern of gene expression in the 3 children's groups was indistinguishable.

**Conclusion:**

The molecular signatures of children of CMS patients show impaired adaptation to hypoxia. At altitude children of CMS fathers had defective coupling between glycolysis and mitochondria TCA cycle, which may be a key mechanism/biomarker for adult CMS. Early biologic markers of disease susceptibility in Andeans might impact health services and social planning.

## Background

Human beings are considered hypoxia-sensitive; however, evidence for different adaptive strategies by various highland populations in coping with their hypoxic environment is abundant. Tibetans, for example, with a longer history of high altitude residence, seem better adapted than Andeans. This is implied by different steps in the oxygen transport cascade [1,2]. Thus, average resting ventilation and arterial oxygen saturation (SaO_2_) are higher in Tibetans than in Aymaras and Quechuas of the Andes. In addition, Tibetans show lower frequency of intrauterine growth retardation, better neonatal oxygenation, lower pulmonary artery pressure and resistance, and lower frequency of chronic mountain sickness (CMS); a sudden loss of adaptation to their highland hypoxic abode appearing first in men in early midlife and in post-menopausal women [3,4]. In Ethiopians, SaO_2 _and haemoglobin values in high altitude residents similar to those of sea level subjects have also been reported [5]. These populations have been residents of the high altitude regions of the world for different lengths of time [6,7]. Natural selection may have equipped Tibetans and Ethiopians, who have resided at altitude for longer times, with molecular adaptations that have not, as yet, made their impact in the Andes. Moreover, not all subjects within a population of highlanders have similar susceptibilities to CMS, and a tendency to familial clustering of CMS in Andean populations has also been noted [8-10].

We hypothesized that, CMS patients and their children, who do not, as yet, have CMS by clinical criteria, may nevertheless, have suggestive molecular signatures that hint at an inability to cope with the metabolic demands of ambient hypoxia. This could be discernible in the impaired transformation of glycolysis-derived pyruvate into Acetyl CoA for efficient ATP production in mitochondrial TCA cycle and by the inadequate expression and activity of key enzymes controlling the coupling between glycolysis and TCA cycle in the face of hypoxia. Thus in adult CMS patients and their clinically normal children, the matching of cellular energy demands with efficient ATP production, in ambient hypoxia, might be impaired, eventually, leading to cellular energy "failure" in the patients[11]. This deficiency might already be discernable, to some extent, at a younger age.

Adaptation to chronic hypoxia in children such as physical growth, oxygen saturation, haemoglobin concentration, respiratory rate, and other cardiopulmonary modifications have been documented. [12-15]. To date no correlations of phenotypic changes with molecular signatures in children of Andean highlanders have been reported. Preliminary data from the highlands do suggest, however, that perinatal exposure to chronic hypoxia increases susceptibility to the later development of maladaptation to life in chronic hypoxia such as CMS. [16]. We hypothesized that the molecular signatures of children might serve as biomarkers for the occurrence of CMS in adulthood.

We focused on oxygen-responsive genes and on those involved in glycolytic and mitochondrial metabolic pathways [17,18].

## Methods

### Design

A cross-sectional study was performed in three groups; children of high altitude natives with CMS; children of high altitude controls, and sea level children without Andean ancestry. Absence of Andean ancestry was defined as absence of parents and grandparents native to high altitude. Ten children were recruited in each high altitude group and 20 in the sea level group. For each adult one child was studied. The children were matched by age and sex.

The protocol was approved by the Ethics Committee of Cayetano Heredia University, Lima, Peru. Before performing any procedure, a written informed consent was obtained from parents. Each child also gave separate written assent.

### Inclusion criteria

Children native to and resident at Cerro de Pasco, aged 5 to 17 years, that did not drink regularly herbal infusions that might reduce red cell mass were included.

### Exclusion criteria

Children with significant acute or chronic clinical conditions or those who had visited altitudes below 2000 m in the last 3 months were excluded.

### Study procedures

High altitude children were studied in Cerro de Pasco first, and then transferred to Lima. Sea level natives were studied in Lima.

A clinical history, physical examination, weight and height measurements, and arterial oxygen saturation (pulse oximetry, SaO_2_) were obtained. Information on relevant current and past diseases, use of cytotoxic drugs or herbal infusions considered by natives as effective for reducing polycythaemia, and history of descent to altitudes below 2000 m. were noted.

Blood samples were taken for haemoglobin and haematocrit determinations, and for RNA assays. Eight milliliters were drawn from children in Cerro and the same volume was taken in Lima. Haemoglobin was determined through spectrophotometry and haematrocrit through a standard capillary technique. At sea level, blood was taken from high altitude subjects within 1 hour after arrival to Lima for gene expression determination.

### Determination of gene expression through quantitative real-time PCR

For quantitative real-time PCR determination, the total RNA of white blood cells was extracted through PAXgene Blood RNA kit (Qiagen, Germany) [11]. In brief, one microgram of total RNA was reverse transcribed into first-strand cDNA using the RETROscript reverse transcriptase kit and oligo dT primers (Ambion, TX), following the manufacturer's protocols. One microliter of cDNA from the reverse transcriptase reaction was used as the template for quantitative real-time PCR reaction with a final PCR reaction volume of 25 ml, with the 59 and 39 gene specific PCR primer concentrations at 100 nM each. PCR primers were designed using Primer3 software (Whitehead Institute, MIT, Massachusetts, USA) according to the coding sequences of each gene. Quantification of mRNA expression was performed by triplicate using the SYBR Green SuperMix (BioRad, California, USA) and a 2-step PCR reaction procedure, performed on the MyiQ Single Color Real-Time PCR Detection System (BioRad, California, USA). After the initial denaturation at 95°C for 3 minutes, 45 cycles of primer annealing and elongation were conducted at 58°C for 45 seconds, followed by denaturation at 95°C for 10 seconds. Fluorescent emission data were captured, and mRNA levels were quantified using the threshold cycle value. To compensate for variations in input RNA amounts and efficiency of reverse transcription, data for each target gene mRNA of each sample were normalized by reference to the data obtained for the house keeping HPRT (GenBank accession number X62085) determined from the same sample. Each real-time PCR assay was repeated twice.

Definition and explanation of terms related to gene expression are detailed as an appendix (See additional file 1).

### Data analysis

To assess the impact of specific genes on chronic mountain sickness-score (CMS-sc), using a sliding scale (continuous), we constructed an "impact table" for the combined (CMS & Controls) Cerro de Pasco groups of fathers at altitude (Cerro de Pasco, 4338 m.) and at sea level (Lima, 150 m.). The impact table was constructed using CMS-sc of the fathers as a continuous variable and consists of the effect sizes (standardized beta) for each gene in univariate linear regression in the first columns, the effect sizes for each gene adjusting for the best univariate predictor (HPH3 at both altitudes) in the middle columns, and the residual effect size of HPH3 adjusting for each of the other genes in the final columns. The STB is the standardized estimate for the parameter estimate of an explanatory variable in the logistic regression model and is computed by multiplying the estimate by the sample standard deviation for the explanatory variable and dividing by π/√ 3. In addition to the comparison among high altitude groups, the Lima control subjects were compared to the high altitude groups, while in Lima.

Gene expression data were log transformed to obtain symmetric distributions for analysis and for graphical purposes. For comparing means of more than two groups at high altitude and at sea level, ANOVA test was run. The gene expression variables that best predicted the CMS classification of 10 Cerro de Pasco fathers with CMS and 10 Cerro de Pasco control-fathers were obtained by considering 1 and 2 variable models in stepwise and "all subsets" logistic regression, sensitivity and specificity using median cut scores, and the effect sizes (standardized beta) in univariate and bivariate logistic regressions at sea level (Lima) and at altitude (Cerro de Pasco, 4338 m). The best two gene model using expressions at sea level (Lima) in the fathers was LDHA based on sensitivity (90%) and specificity (100%) and PDP2 based on effect size (standardized beta) in logistic regression [11]. Since lactate dehydrogenase A (LDHA) was not measured at high altitude (Cerro), PDK3 was used, based on having the highest correlation with LDHA at Lima. The "niche" scatterplots show the two predictor genes selected as best at each altitude for the children.

All the analyses were run using SAS 9.1 and SPSS 13.0.

## Results

Children from all three groups were comparable in weight, height, blood pressure, respiratory rate, and heart rate. Sea level children were somewhat younger than those from high altitude (Table 1). As expected, haemoglobin and haematocrit were significantly lower and SaO_2 _significantly higher in sea level children than in any of the high altitude groups (Table 1).

**Table 1:** Demographic and clinical characteristics of children by groups

	**SL children**	**HA control children**	**CMS children**
**Age (yrs)**	9.91(3.15)*	13.05(3.35)	12.24(2.65)
**Weight (kg)**	38.45(13.53)	37.98(10.49)	35.87(10.67)
**Height (cm)**	132.45(20.41)	140.60(14.37)	140.70(17.46)
**Hb(g/dL)**	12.74(0.84)**	15.44(1.02)	15.69(1.42)
**Hct (%)**	38.73(2.52)**	47.08(3.09)	47.45(4.29)
**SaO**_2_	98.95(2.11)*	94.75(5.56)	94.30(6.34)
**HR (beats/min)**	74.30(12.06)	69.35(10.82)	75.05(18.22)
**RR (resp/min)**	23.50(5.46)	22.50(4.35)	23.80(5.06)
**SBP (mmHg)**	95.00(13.95)	97.00(8.01)	92.00(8.94)
**DBP (mmHg)**	57.75(8.66)	58.00(8.94)	57.50(7.69)

SL: sea level; HA: high altitude; CMS: children from subjects affected by CMS. Difference between groups (ANOVA): *p < 0.05; **p < 0.001

### HIF-regulated genes

The impact table shows the predicting power of specific hypoxia-related genes on CMS in the fathers of Andean children (Table 2). Note that the genes were assayed in children, whereas the CMS scores were those of their fathers.

**Table 2:** Impact table to show the predicting power of specific hypoxia-related genes on CMS (CMS-score ≥ 12) in the fathers of Andean children

**In Children**				**Adjust for HPH3**			**Adjust HPH3**	
**At Cerro**	**STB**	**P**	**Cerro**	**STB**	**P**	**Cerro**	**STB**	**P**
log_EPO	-0.07	0.79	log_EPO	-0.36	0.32	log_HPH3	0.88	0.05
log_HPH1	0.02	0.94	log_HPH1	0.06	0.84	log_HPH3	0.75	0.07
log_HPH2	0.21	0.44	log_HPH2	0.20	0.53	log_HPH3	0.75	0.07
**log_HPH3**	**0.74**	**0.07**						
log_VEGFC	-0.05	0.85	log_VEGFC	-0.38	0.27	log_HPH3	0.96	0.05
log_PDK1	-0.30	0.38	log_PDK1	-2.69	0.18	log_HPH3	2.82	0.08
log_PDP1	0.16	0.55	log_PDP1	-0.36	0.38	log_HPH3	1.00	0.06
log_HIF1A	0.67	0.07	log_HIF1A	0.51	0.19	log_HPH3	0.61	0.17
log_HIF1B	-0.43	0.24	log_HIF1B	-1.28	0.07	log_HPH3	1.84	0.08
log_HIF2A	0.61	0.08	log_HIF2A	0.78	0.13	log_HPH3	1.00	0.11
log_HIF3A	0.01	0.98	log_HIF3A	-0.17	0.62	log_HPH3	0.77	0.06
log_POK2	0.02	0.94	log_POK2	-0.20	0.53	log_HPH3	0.80	0.06
log_PDP2	0.06	0.83	log_PDP2	-0.18	0.58	log_HPH3	0.80	0.06
log_PDK3	-0.37	0.26	log_PDK3	-0.91	0.04	log_HPH3	1.39	0.04
log_PDK4	-0.21	0.45	log_PDK4	-0.20	0.51	log_HPH3	0.73	0.07
log_PDHE1A1	-0.25	0.39	log_PDHE1A1	-0.98	0.06	log_HPH3	1.54	0.05
log_GADPH	-0.20	0.48	log_GADPH	-1.01	0.08	log_HPH3	1.62	0.05
log_EPOR	0.24	0.40	log_EPOR	-0.38	0.43	log_HPH3	1.02	0.07
log_GLUT1	-0.15	0.59	log_GLUT1	-0.53	0.15	log_HPH3	1.05	0.05
log_LDHA	missing							
log_CATD	missing							
								
				**Adjust for LDHA**			**Adjust LDHA**	
**At Lima**			**Lima**	**STB**	**P**	**Lima**	**STB**	**P**
log_EPO	-0.13	0.61	log_EPO	0.13	0.70	log_LDHA	-0.64	0.09
log_HPH1	0.29	0.28	log_HPH1	0.45	0.20	log_LDHA	-0.69	0.07
log_HPH2	-0.38	0.18	log_HPH2	-0.20	0.51	log_LDHA	-0.50	0.16
log_HPH3	0.12	0.67	log_HPH3	0.40	0.34	log_LDHA	-0.84	0.08
log_VEGFC	missing		log_VEGFC					
log_PDK1	-0.27	0.34	log_PDK1	0.17	0.68	log_LDHA	-0.68	0.10
log_PDP1	-0.51	0.09	log_PDP1	-0.28	0.44	log_LDHA	-0.40	0.31
log_HIF1A	0.18	0.50	log_HIF1A	0.48	0.20	log_LDHA	-0.76	0.05
log_HIF1B	-0.26	0.32	log_HIF1B	0.11	0.76	log_LDHA	-0.65	0.12
log_HIF2A	-0.21	0.45	log_HIF2A	0.30	0.47	log_LDHA	-0.69	0.07
log_HIF3A	0.29	0.28	log_HIF3A	0.45	0.20	log_LDHA	-0.77	0.08
log_POK2	-0.33	0.24	log_POK2	-0.16	0.64	log_LDHA	-0.52	0.14
log_PDP2	-0.41	0.17	log_PDP2	-0.01	0.99	log_LDHA	-0.57	0.25
log_PDK3	-0.30	0.27	log_PDK3	0.00	1.00	log_LDHA	-0.58	0.14
log_PDK4	-0.32	0.27	log_PDK4	-0.37	0.31	log_LDHA	-0.61	0.09
log_PDHE1A1	-0.27	0.31	log_PDHE1A1	0.68	0.24	log_LDHA	-1.20	0.07
log_GADPH	0.12	0.67	log_GADPH	0.40	0.34	log_LDHA	-0.84	0.08
log_EPOR	0.33	0.25	log_EPOR	1.14	0.08	log_LDHA	-1.21	0.03
log_GLUT1	missing							
**log_LDHA**	**-0.58**	**0.09**						
log_CATD	-0.46	0.13	log_CATD	-0.09	0.85	log_LDHA	-0.51	0.30

In univariate linear regression model, HPH3 (assayed in the children) was found to be the most significant predictor of CMS-sc in the fathers (Table 2, in bold font). Adjusting for HPH3 eliminates the impact of the other genes on the CMS-sc (columns "adjusted for HPH3"). However, adjusting HPH3 for each of the remaining genes, assayed here, strengthens the impact of HPH3 (columns "adjusted HPH3"). This supports the importance of HPH3 as measured in the children, in predicting CMS-sc of their fathers at altitude. However, in Lima (normoxia) in the univariate linear regression model, LDHA was found to be the most significant predictor of CMS-scores of the fathers. But, because there were no significant differences in expression levels in the 3 children's groups at Lima, the impact of LDHA was weakened (columns "adjusted LDHA") in normoxia.

Hypoxia Inducible Factor 3 is a prolyl hydroxylase (HPH), an enzyme that allows oxygen tension to control HIF-alpha protein levels; LDHA is involved in "buffering" pyruvate by reversibly converting to lactate. It favors formation of lactate, when pyruvate builds up too quickly or when there is a block in pyruvate metabolism due to pyruvate dehydrogenase inhibition or lack of sufficient oxygen.

Genes directly regulated by HIF showed lower expression levels (EPO, HPH1, HIF-1 alpha, HIF-1 beta, HIF-2 alpha, HIF-3 alpha, HPH1, HPH3, and EPOR) in the sea level children group than in both of the high altitude groups when their samples were collected at high altitude. By contrast, all genes were expressed at similar levels for all 3 groups at sea level, except for EPOR, which was higher in HA controls (Table 3). Gene expression did not differ significantly between HA controls and HA children of fathers with CMS while they were studied at high altitude (Table 3).

**Table 3:** Expression of genes regulated by HIF, at high altitude and at sea level

	**CMS children at HA**	**CMS children at SL**	**HA control at HA**	**HA controls at SL**	**SL children**
**log EPO**	0.042(0.029)*	-1.979(0.189)	0.040(0.025)	-1.939(0.177)	-1.793(0.502)
**log HIF-1 alpha**	-6.059(0.565)*	-2.577(0.229)	-6.647(0.453)	-2.699(0.538)	-2.491(0.657)
**log HIF-1 beta**	-3.069(0.385)*	0.192(0.143)	-2.865(0.200)	0.258(0.155)	0.191(0.252)
**log HIF-2 alpha**	-3.763(0.369)*	-1.183(0.172)	-4.062(0.342)	-1.086(0.376)	-0.989(0.376)
**log HIF-3 alpha**	-5.430(0.432)*	-2.980(1.125)	-5.422(0.225)	-3.463(0.848)	-2.963(0.844)
**log HPH1**	-0.245(2.880)*	-2.980(1.125)	-0.088(2.885)	-3.463(0.848)	-2.963(0.844)
**log HPH2**	1.063(2.352)	0.532(0.136)	-0.149(2.357)	0.613(0.124)	0.447(0.207)
**log HPH3**	-5.545(0.673)*	-3.351(1.528)	-6.166(0.499)	-3.646(1.431)	-2.773(1.701)
**log EPOr**	-2.981(0.340)*	0.127(0.159)	-3.056(0.204)	0.026(0.217)	-0.029(0.189)

Difference between groups (ANOVA): *p < 0.001

### Genes involved in glycolysis and in the Krebs cycle

Gene expression of PDK1, PDK2, PDK3, PDK4, PDP1, PDP2, PDHE1A1, and GADPH was higher in sea level children than in their high altitude counterparts when they were examined in their native high altitude environment (Table 4). Whereas, at sea level, children of fathers with CMS showed similar gene expression levels to HA controls and sea level children (Table 4).

**Table 4:** Expression of genes involved in glycolisis, Krebs cycle, and related genes – at high altitude and at sea level

	**CMS children at HA**	**CMS children in Lima**	**HA control at HA**	**HA controls at SL**	**SL children**
**log PDK-1**	-3.591(0.459)*	0.223(0.176)	-3.419(0.149)	0.299(0.178)	0.253(0.243)
**log PDK-2**	-2.869(0.172)*	0.203(0.111)	-2.858(0.167)	0.281(0.173)	0.171(0.240)
**log PDK-3**	-3.114(0.414)*	0.551(0.127)	-2.918(0.206)	0.620(0.147)	0.446(0.222)
**log PDK-4**	-4.536(0.353)*	-0.564(0.310)	-4.409(0.387)	-0.402(0.331)	-0.833(0.368)
**log PDP-1**	-2.997(0.217)*	0.132(0.141)	-3.019(0.237)	0.238(0.113)	0.030(0.214)
**log PDP-2**	-3.750(0.444)*	-0.678(0.201)	-3.776(0.147)	-0.576(0.091)	-0.604(0.196)
**log PDHE1A1**	-3.357(0.387)*	0.003(0.140)	-3.193(0.308)	0.070(0.154)	0.001(0.206)
**log GADPH**	-1.835(0.304)**	-3.351(1.528)	-1.744(0.183)	-3.646(1.431)	-2.773(1.701)

Difference between groups (ANOVA): *p < 0.001; **p < 0.005

### Niche graphs

**Figure 1:**
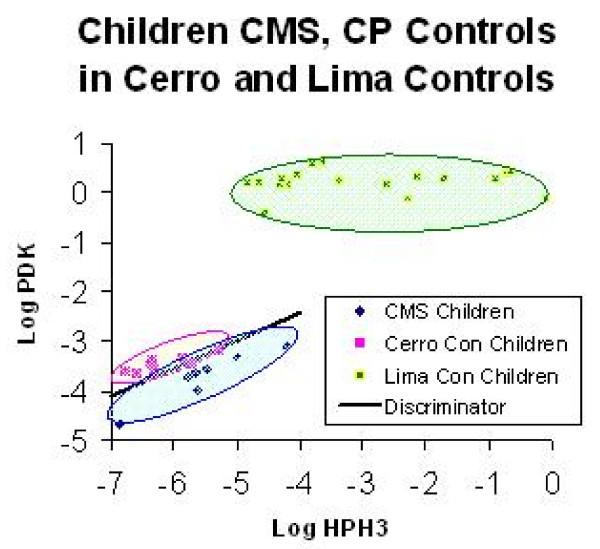
Niche (scatter) plots of gene expression levels in children that predict the CMS status of their fathers.

**Figure 2:**
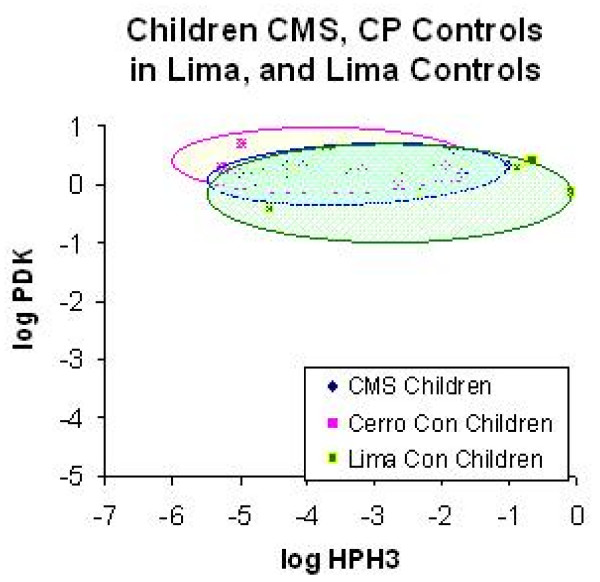
**Niche (scatter) plots of gene expression levels in Lima in all 3 study groups**. CP = Cerro de Pasco, 4338 m. Cerro = Cerro de Pasco. CMS = chronic mountain sickness. Con = controls.

The "niche" scatter plots illustrate the two gene expression levels that best predicted "CMS parenthood" in the children (HPH3 and PDK1). This expression was significantly lower at altitude than at sea level (Fig. 1). Moreover, the children of parents with CMS had even lower expression levels of these genes than HA control children; and they were clearly separated by the discriminator line constructed based on discrimination of the two groups by logistic regression (Fig. 1). The control SL children, at sea level in Lima, have still higher expression levels for HPH3 and PDK1 (Fig. 1). Immediately after descent to sea level, normoxia, all highland children had similar expression levels of HPH3 and PDK1 (Fig. 2).

## Discussion

Chronic mountain sickness causes enormous social problems in the high Andes such as early death of bread winners in usually large families and excessive burdens on already meager health services in the highlands [19]. To anticipate the occurrence of CMS and initiate preventive treatment may contribute to the well being of large numbers of villagers in the Andes and Altiplano. These social concerns have underscored our efforts to determine possible predictors of this disease.

We show, for the first time, using molecular signatures, defined as distinct patterns of gene expression, that children of fathers with CMS, who themselves show no signs of ill health, have distinguishing molecular signatures in their native highland environment (Fig. 1); a possible biomarker of the disease. By contrast, when hypoxia is removed by short sea level sojourn, the molecular signatures in these same highland children are indistinguishable from sea level children, (Fig. 2). We emphasize that no clinical evidence of CMS was detectable in the children, neither in their native highland village, nor during their short stay in Lima, Peru. The later occurrence of CMS, usually a disease of older men and post-menopausal women, may, therefore, be predictable during childhood, with important public health implications.

High altitude children had low expression of HPH3 and PDK1 in their native environment. Whereas HPH3 allows oxygen tension to control HIF-alpha protein levels; PDK1 is an enzyme involved in "aerobic glycolysis" (the Warburg effect). This enzyme supports a metabolic pattern seen in many hypoxia adapted tissues. Low expression of these two enzymes was a characteristic feature of children fathered by CMS patients when studied in their native ambient hypoxia (Fig. 1). But the removal of ambient hypoxia for 1 hour in Lima was sufficient to increase expression of these genes to levels comparable to those found in children of sea level fathers who have no altitude ancestry (Fig. 2).

How could HPH3 and PDK1 expression levels predict chronic mountain sickness later in life? Hypoxia, such as is inescapable in the highlands, leads to the formation of free radicals and oxidants [20]. These chemical species, by-products of mitochondrial metabolism, include the superoxides (O_2_^-^), peroxides (H_2_O_2_) and hydroxyl radicals (OH^-^) are collectively designated as reactive oxygen species (ROS) [21]. However, these molecules are also crucial for the maintenance of homeostasis, especially in the face of hypoxia. The ROS abundantly formed during hypoxia modify proteins, allowing them to detect oxidants and maintain homeostasis, as has recently been shown for the guanosine 3',5'-monophosphate cGMP-dependent protein kinase PKG 1α isoform, a direct peroxide (H_2_O_2_) sensor [22]. In addition, deficient peripheral PDK1 and HPH3 mRNA expression at altitude in children of CMS parents attests to a defective coupling between glycolysis and mitochondria TCA cycle, which may be a key mechanism/biomarker for adult CMS.

Recent studies on hypoxic cells identified critical adaptations that could prevent hypoxia-induced increases in ROS production and thus forestall ROS damage to cellular proteins. Amongst those adaptations is increased expression of PDK1 [17], and HPH3 may act in a similar fashion. Thus the childhood reduction of hypoxia-induced ROS accumulation at altitude, by appropriate up-regulation of PDK1 and HPH3, to levels we found in control children, might prevent time induced accumulation of ROS which ultimately may lead to the clinical development of the CMS syndrome in the children fathered by CMS patients.

The maintenance of homeostasis, the ability to maintain a steady state in the face of hypoxic stress, is fundamental to adaptation for life anywhere, including life in chronic hypoxia. Failure to upregulate genes that help maintain cellular homeostasis in hypoxia, as we found here, may place these children at an evolutionary disadvantage. Not surprisingly such disadvantage, if based on genetic differences, might be evident early in life.

Gene expression levels, as measured here, are the result of many factors that act on the DNA sequence. Epigenetic influences, a set of heritable DNA or protein changes not involving DNA mutations, are known to affect gene expression [23,24]. In addition, early environmental factors acting during embryonic, fetal, or early postnatal periods may also modify gene expression [12,16,23].

We must acknowledge some methodological constraints. We studied our subjects in the field and thus strict laboratory procedures were usually unattainable. In addition, although larger numbers of subjects in each study group were desirable these could not be assembled because of time limitations at altitude for sea level researchers and especially because molecular studies were combined with clinical evaluations in the same individuals. The research station of the Universidad Peruana Cayetano Heredia in Cerro de Pasco, Peru maintains a list of CMS patients and controls. We used this list to recruit our subjects in Peru. Because women are protected from CMS until the menopause, the list contains mainly men. Thus, the results of our earlier studies had unavoidable gender bias. Subsequently, the gender bias imposed on us previously constrained our selection. Therefore we used fathers only for CMS scoring purposes.

In previous field studies we found that exposure to 1 hour of normoxia at sea level, or 1 hour of hyperoxia at the resident altitude of highlanders [11] was sufficient to change gene expression in white cells [25], which predicated the short exposure times of highland children in Lima.

## Conclusion

We designed this study to measure gene expression that may predict the later occurrence of CMS. Regardless of how such biomarkers may have been induced, by epigenetic influences, evolutionary pressures or failure to respond to hypoxic stress, our findings point to an important role of particular genetic signatures in highland children for the later development of CMS. We anticipate that use of such biomarkers in planning for health promotion and social justice in the highlands of Peru might benefit the future of highland children in the Andes.

## Competing interests

The authors declare that they have no competing interests.

## Authors' contributions

LH, OA and AV conceived and designed the experiments. GX performed gene expression analyses. CQ, OA and LH analyzed the data. AV, MRC, JG, and GX contributed with logistic and clinical support, and with selection of genes of interest. LH wrote the paper, and all other authors reviewed the manuscript and contributed with comments. All authors saw and approved the final version.

## Funding

New Mexico Health Enhancement and Marathon Clinics Research Foundation Albuquerque NM USA. NIH Ro1 NS37814 to Ajay Verma.

## Role of the funding source

The sponsors of the study had no role in the study design; collection, analysis, or interpretation of data; writing of the paper; or the decision to submit the paper for publication. The corresponding author had full access to all the data in the study and had final responsibility for the decision to submit for publication.

## Pre-publication history

The pre-publication history for this paper can be accessed here:



## Supplementary Material

Additional file 1 **Supplementary file 1.** Definition and explanation of terms related to gene expression. Click here for file
