# Supplementary material for: Abnormal energy regulation in early life: childhood gene expression may predict subsequent chronic mountain sickness
Source: BMC Pediatr. 2008 Oct 27;8:47. doi: 10.1186/1471-2431-8-47 (PMC2582028; doi:10.1186/1471-2431-8-47)
Supplement: Additional file 1 — Supplementary file 1. Definition and explanation of terms related to gene expression. [file 1471-2431-8-47-S1.doc]

**Supplementary file 1**

**Molecular signature:** A distinct pattern of gene expression.

***EPO:*** Erythropoietin gene. Involved in erythropoiesis. Its transcriptional control is mediated by HIF..

**EPOR:** Erythropoietin receptor.

**HPH1,HPH2, HPH3:** HIF prolyl hydroxylases (HPH). Enzymes that allow

oxygen tension to control HIF-alpha protein levels.

**HIF1A, HIF2A, HIF3A, HIF1B:** Composed of alpha and beta subunits. Only the alpha subunit protein levels are regulated by oxygen. HIF1B is used as a dimerizing partner by all three HIF1A’s.

**PDK1, PDK2, PDK3, PDK4:**. Pyruvate dehydrogenase kinases. PDK2 is the

most widely expressed form of PDK. These kinases are involved in "aerobic

glycolysis" (the Warburg effect) by inhibiting pyruvate metabolism and favoring lactate accumulation. They support a metabolic pattern seen in many (but not all) hypoxia adapted tissues, and also in cancer cells and activated immune cells.

**PDP2,1**= Phosphatases that de-phosphorylate the E1 alpha subunit of pyruvate

dehydrogenase. The enzymes encoded by this gene attempt to reverse the dephosphorylation activity of PDKs and promote pyruvate entry into the Krebs cycle.

**PDHE1A1**= the gene encodes the E1 alpha subunit of pyruvate dehydrogenase

(PDH), which is the regulatory component of PDH and the target of PDKs and

PDPs.

**VEGFC:** Vascular endothelial growth factor, the C-form was measured.

**GAPDH**= Glyceraldehyde phosphate dehydrogenase. Regulated by HIF. GAPDH has a central position in the glycolytic cascade.

**GLUT1**= Glucose transporter protein 1. GLUT1 is known to be one glucose

transporter sensitive to hypoxia and HIF1; it is widely expressed in most tissues.

**LDHA**= Lactate dehydrogenase A. LDHA is involved in "buffering" pyruvate by

reversibly converting to lactate. It favors formation of lactate, when pyruvate

builds up too quickly or when there is a block in pyruvate metabolism due to

pyruvate dehydrogenase inhibition or lack of sufficient oxygen.

**CATD**= Cathepsin-D. CATD is a protease involved in extracellular matrix

modification and tissue invasion. It is a HIF1 regulated gene relevant to the

biology of many invasive cells but has other functions as well.
